# Clinical Audit and Evaluation of Dementia Access and Wait Times

**DOI:** 10.1192/bjo.2026.11844

**Published:** 2026-06-30

**Authors:** Olusegun Sodiya, Ifechukwu Iloekwe, Mani Krishnan

**Affiliations:** Tees, Esk, Wear Valleys NHS Foundation Trust, Darlington, United Kingdom

## Abstract

**Aims:**

This clinical audit aimed to assess and quantify patient wait times across all localities within the TEWV Trust, benchmarking these durations against Trust and National averages. Timely care for individuals with dementia is critical, not only for patients and their families but also for broader community health outcomes. Early diagnosis is essential for providing customized support, facilitating effective treatment options, enabling proactive planning, and preventing crises. Reducing the wait time between referral to a Memory Assessment Service (MAS) and the subsequent diagnosis and care plan is crucial, as delays can significantly burden the NHS and adversely affect the long-term mental health of patients and their caregivers. According to the National Audit of Dementia for 2023/2024, wait times have risen sharply from 124 days in 2021 to 151 days, with only 10% of patients obtaining a diagnosis within six weeks of referral.

**Methods:**

The audit was conducted over a four-week period from September 28 to October 28, 2024. A random sampling method was employed to select 149 patients referred to 15 memory services throughout the TEWV Trust. Data were collected through the analysis of referral letters, initial assessments, diagnostic appointments, case notes, and correspondence from CITO. Analysis was performed using a custom tool developed in Excel.

**Results:**

The analysis yielded average access times, diagnostic wait times, and overall wait times ranging from 30 to 207 days, 3 to 147 days, and 92 to 323 days, respectively. Notably, none of the Mental Health Service for Older People (MHSOP) services within the TEWV Trust met the national benchmark for overall wait times of six weeks. Furthermore, only 25% of the services succeeded in providing a diagnosis within the 6 to 18-week timeframe, while the remaining 75% experienced wait times extending to 18-52 weeks. A comparative analysis revealed that the average wait times (access, diagnostic, and overall) within the Trust exceeded those of the National averages.

**Conclusion:**

The audit underscored substantial discrepancies between the various memory services’ performance and the National recommendations of a six-week wait. Among the services assessed, only one was marginally compliant with a 92-day mark, falling short of the National benchmark of 42 days. This evaluation not only identified critical challenges faced by the services but also presented a series of recommendations to address these gaps.

